# The Identification and Analysis of mRNA–lncRNA–miRNA Cliques From the Integrative Network of Ovarian Cancer

**DOI:** 10.3389/fgene.2019.00751

**Published:** 2019-08-21

**Authors:** You Zhou, Xiao Zheng, Bin Xu, Wenwei Hu, Tao Huang, Jingting Jiang

**Affiliations:** ^1^Department of Tumor Biological Treatment, The Third Affiliated Hospital of Soochow University, Changzhou, China; ^2^Jiangsu Engineering Research Center for Tumor Immunotherapy, Changzhou, China; ^3^Institute of Cell Therapy, Soochow University, Changzhou, China; ^4^Shanghai Institute of Nutrition and Health, Shanghai Institutes for Biological Sciences (CAS), Shanghai, China

**Keywords:** variance inflation factor regression, mRNA–lncRNA–miRNA cliques, regulatory network construction, ovarian cancer, functional regulator

## Abstract

Ovarian cancer is one of the leading causes of cancer mortality in women. Since little clinical symptoms were shown in the early period of ovarian cancer, most patients were found in phases III–IV or with abdominal metastasis when diagnosed. The lack of effective early diagnosis biomarkers makes ovarian cancer difficult to screen. However, in essence, the fundamental problem is we know very little about the regulatory mechanisms during tumorigenesis of ovarian cancer. There are emerging regulatory factors, such as long noncoding RNAs (lncRNAs) and microRNAs (miRNAs), which have played important roles in cancers. Therefore, we analyzed the RNA-seq profiles of 407 ovarian cancer patients. An integrative network of 20,424 coding RNAs (mRNAs), 10,412 lncRNAs, and 742 miRNAs were construed with variance inflation factor (VIF) regression method. The mRNA–lncRNA–miRNA cliques were identified from the network and analyzed. Such promising cliques showed significant correlations with survival and stage of ovarian cancer and characterized the complex sponge regulatory mechanism, suggesting their contributions to tumorigenicity. Our results provided novel insights of the regulatory mechanisms among mRNAs, lncRNAs, and miRNAs and highlighted several promising regulators for ovarian cancer detection and treatment.

## Introduction

Ovarian cancer (OC) ranks the fifth leading cause of cancer mortality among gynecologic malignancies in women (Torre et al., 2018). According to the SEER registry, 22,240 new cases and 14,070 deaths of ovarian cancer have been estimated in the United States in 2018. Ovarian cancer, encompassing various tumors of ovarian origin, is a typical example of heterogeneous disease and classified into three categories based on the affected cells: epithelial cells, germ cells, and stromal cells, of which epithelial ovarian cancer (EOC) is the most common cause of death within gynecologic cancer (Vetter and Hays, 2018). Due to the insidious nature of ovarian cancer, manifesting with little to no clinical symptoms in the early period, 80% of patients were found in stage III or IV, which explains the low 5-year survival rate and poses significant therapeutic challenges (Torre et al., 2018). Although great progress has been made in screening methods such as transvaginal ultrasonography as well as serum biomarker CA125 and HE4 for early detection of ovarian cancer, diagnostic and treatment limitations still exist because of low sensitivity, high expenses, and/or inconvenience. Thus, it is imperative to identify new risk genes and their regulatory network in order to elucidate ovarian carcinogenetic mechanisms through genetic research.

A decade-long interest in the cross talk between coding RNAs (mRNAs) and noncoding RNAs has unveiled multilayer regulatory circuitry of cells including chromatin remodeling, protein stability, transcription, and mRNA turnover; the dysregulation of which could also lead to the occurrence of cancer. Increasing studies have demonstrated that microRNAs (miRNAs), which are small noncoding RNAs (∼22 nts), and long noncoding RNAs (lncRNAs), which are usually longer than 200 nucleotides, mutually regulated their expression levels and worked jointly to control the expression of mRNAs (Braconi et al., 2011; Fan et al., 2013; Martens-Uzunova et al., 2014; Liu et al., 2017a). Conversely, mRNAs also impose their influences on the expression of noncoding RNAs in a variety of ways (Helwak et al., 2013; Ribeiro et al., 2014; Sundaram and Veera Bramhachari, 2017). For example, a muscle-specific lncRNA linc-MD1 elevated the expression of MAML1 and MEF2C mRNAs by preventing miR-133 and miR-135 from binding to their targets (Cesana et al., 2011; Legnini et al., 2014). Linc-ROR has been reported to function as a key competing endogenous RNA to partially sequester miR-145-5p, which was activated by Nanog, Oct4, and Sox2, forming a feedback loop with core transcription factors and miRNAs to regulate embryonic stem cell maintenance and differentiation (Wang et al., 2013). The widely studied lncRNA HOTAIR acted as a scaffold to link several RNA-binding proteins including Ataxin-1 and Snurportin-1, whose stability was decreased by miR-34a in the human prostate cancer cells (Chiyomaru et al., 2013; Yoon et al., 2013). Such interactions are called mRNA–lncRNA–miRNA triplets; the aberration of which could destroy the gene expression patterns and promote tumorigenesis (Gao et al., 2015; Imig et al., 2015; Wang et al., 2015a).

With the development of Next Generation Sequencing (NGS), all kinds of RNAs, such as lncRNAs, miRNAs, mRNAs, Piwi-interacting RNAs (piRNAs), and circular RNAs (circRNAs), can be easily quantified. The complex regulatory mechanism among them can be investigated with advanced network analysis. For ovarian cancer, The Cancer Genome Atlas (TCGA) (The Cancer Genome Atlas Research Network et al., 2013) is a valuable dataset with the mRNA, lncRNA, and miRNA expression profiles of 407 patients. To construct the mRNA–lncRNA–miRNA regulatory network and the cancer-related mRNA, lncRNA, and miRNA triplets, we adopted variance inﬂation factor (VIF) regression method (Liu et al., 2017a). A comprehensive genome-wide mRNA, lncRNA, and miRNA regulatory network of ovarian cancer was built, and the substructures of mRNA–lncRNA–miRNA cliques were discovered on the network. These triplets were further filtered by associations with survival and ovarian cancer stage. At last, nine triplets were analyzed in detail. The mechanisms revealed by this work may help understand the tumorigenesis and may develop new treatment for ovarian cancer.

## Methods

### Ovarian Cancer RNA-Sequencing Datasets

The RNA-sequencing data of ovarian cancer are downloaded from Akrami et al. (2013) at http://larssonlab.org/tcga-lncrnas/datasets.php. It included the expression levels of 20,462 protein coding mRNAs, 10,419 lncRNAs, and 742 miRNAs in 407 ovarian cancer patients. The summary of this ovarian cancer dataset is shown in **Table 1**. As described by Akrami et al. (2013), the expression levels of these transcripts were quantified as RPKM (reads per kilobase per million mapped reads) by normalizing the mRNA length and library size after the read counts were generated using TopHat (Kim et al., 2013) and HTSeq-count (Anders et al., 2015). To enable the log2 transformation, the zero values of the expression data were replaced with the minimum nonzero RPKM values. The TCGA sample IDs and their clinical information were listed in **Supplementary Material 1**. Within the 407 samples, four samples did not have stage information, 20 samples were in stage II, 323 samples were in stage III, and 60 samples were in stage IV. The sample distribution was consistent with the incidences of ovarian cancer: most of them were in stages III and IV (Torre et al., 2018).

**Table 1:** Number of transcripts in expression data, constructed network, triplets of the 407 ovarian cancer patients.

	mRNA	lncRNA	miRNA	Total
**Expression**	20,462	10,419	742	31,623
**Network**	16,667	4,796	207	21,670
**Triplets**	633	1,184	169	1,986

## The mRNA–lncRNA–miRNA Network Construction Based on Variance Inflation Factor Regression

Previous studies have shown that miRNAs and lncRNAs can interact with each other and regulate the expression of mRNAs jointly (Braconi et al., 2011; Fan et al., 2013; Martens-Uzunova et al., 2014; Liu et al., 2017a). To get a genome-wide view of such complex regulations among miRNAs, lncRNAs, and mRNAs, we adopted a method called variance inflation factor (VIF) regression (Lin et al., 2011) to construct the mRNA–lncRNA–miRNA network of ovarian cancer.

Since there were 31,623 transcripts in total, including 20,462 protein coding mRNAs, 10,419 lncRNAs, and 742 miRNAs, for each regression model that corresponded to a transcript, there will be 31,622 variables that may regulate it. It will be a very large regression model. The computational complexity is extremely high. Fortunately, the VIF algorithm is a highly efficient method and can select the possible regulators of the target RNA in a short time.

The optimization goal of the VIF algorithm is to estimate β that minimizes *l_0_* norm (Lin et al., 2011), the penalized sum of squared errors between actual expression levels of the target transcript and predicted expression levels,

(1) $$\arg\min_{\beta }\left\{{\left\|y-X\beta \right\|}_{2}^{2}+\lambda_{0}\sum_{i=1}^{p}I_{\left\{\beta_{i}\neq 0\right\}}\right\}$$

where ***y*** are actual expression levels of the target transcript in 407 ovarian cancer patients, **x** are the expression levels of the 31,622 candidate regulators, β is the vector of coefficient parameters, λ_0_ is the penalty, *p* is the number of candidate regulators, i.e., 31,622 in this study, $I_{\left\{\beta_{i}\neq 0\right\}}$ is the number of coefficients that are not zero.

If we use the conventional method, there will be 2*^p^* possible combinations of candidate regulators to search for the best β. It is an innumerous space to search. The VIF algorithm takes a greed strategy. First, it evaluates the marginal correlation of each candidate regulator with the target transcript in a pre-sampled small dataset. Then, it searches the optimal candidate regulator subset with t-statistic correction by adding/removing one candidate regulator each time. The algorithm is not only efficient but also accurate. Its accuracy is comparable with the highly recognized regression method, LASSO. We used the R package VIF (Lin et al., 2011) to apply the VIF regression algorithm.

After the regression model was constructed, we used the adjust coefficient of determination *R^2^* to evaluate the goodness-of-fit. It measured how well the predicted value approximates the actual values with adjustment of how complex the regression model was, i.e. how many regulators were included in the model. We only kept the regression models with the adjust *R^2^* greater than 0.8. The target transcript and all the regulators in the optimal subset of each regression model constructed a regulatory association network with edge directions that the regulators regulated the target. Then, all the association networks constructed from selected regression models are combined to a single network.

### The Identification and Analysis of mRNA–lncRNA–miRNA Cliques From the Network

As we mentioned before, the mRNA–lncRNA–miRNA triplet functions together and forms a clique on network. A mRNA–lncRNA–miRNA clique includes three fully connected nodes: one mRNA, one lncRNA, and one miRNA. Such structure has significant biological meanings and worth to be identified and analyzed. We used R package *igraph* (Gabor Csardi, 2006) to find the cliques of mRNA–lncRNA–miRNA triplets on the integrative network constructed with VIF regression. In a clique of mRNA–lncRNA–miRNA, there were regulations between lncRNA and mRNA, miRNA and mRNA, and lncRNA and miRNA. Since the transcriptome data were not time course, it was difficult to decide the direction of regulation accurately. We treated the VIF integrative network as an association network rather than a directional network.

### The Survival Analysis of the RNAs Within the mRNA–lncRNA–miRNA Cliques

There were 406 ovarian cancer patients with both the overall survival time (months) and the survival status. We performed survival analysis using Cox proportional hazards regression model (Andersen and Gill, 1982) on these samples. For each RNA, the patients were divided into two groups: the patients with expression levels smaller than the median and the patients with expression levels greater than or equal to the median. The survival curves of these two groups of patients were plotted as Kaplan–Meier plot. The significance of survival difference between these two patient groups was evaluated with log rank test p value calculated by univariate survival analysis. For each triplet, the log rank p value was calculated using multivariate survival analysis of the mRNA, lncRNA, and miRNA. If the log rank test p value was smaller than 0.05, their survivals were considered as significantly different. The R package survival (https://CRAN.R-project.org/package=survival) was used to perform the survival analysis.

## Results and Discussion

### Construction of the Ovarian Cancer Integrative Network Based on VIF Regression

**Figure 1:**
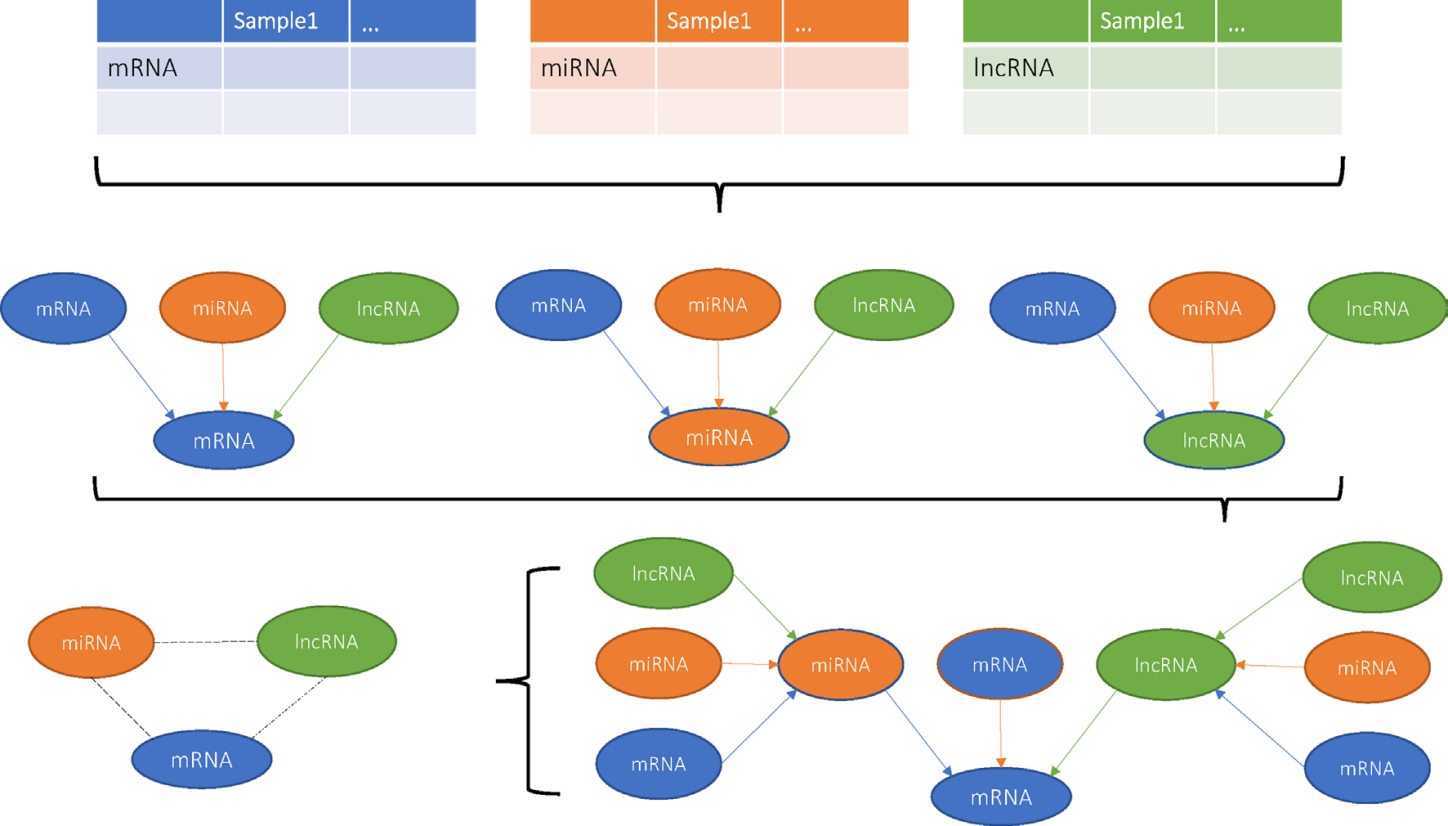
Flowchart of this study.

Previous studies have built various computational models to predict potential disease-related lncRNA/miRNA (Chen and Yan, 2013; Chen and Huang, 2017; Chen et al., 2017; Chen et al., 2018a; Chen et al., 2018b; Chen et al., 2018c) that showed high efficiency, accuracy, and stability. In this study, we firstly introduced cliques incorporating mRNA, lncRNA, and miRNA and constructed their genome-wide integrative network based on VIF regression (**Figure 1**). First, for each target transcript, a regression model was estimated using all the other 31,622 transcripts. The candidate regulators could be mRNA, lncRNA, or miRNA. Then, we filtered the 31,623 VIF regression models with cutoff of adjusted R^2^ greater than 0.8. Finally, we combined the filtered highly possible regression models to obtain a complex integrative network that was given in **Supplementary Material 2**. It had 381,085 regulations between 21,670 transcripts that included 16,667 coding mRNAs, 4,796 lncRNAs, and 207 miRNAs as shown in **Table 1**. It can be seen that about 81.5% measured coding mRNAs, 46.0% measured lncRNAs, and 27.9% measured miRNAs played roles in the integrative network. The lncRNAs may have played more functional regulation roles than miRNAs.

### Identification of mRNA–lncRNA–miRNA Cliques Based on Network Structure Analysis

We analyzed the graph structure of ovarian cancer integrative network using R package *igraph* (Gabor Csardi, 2006) and identified 7,311 mRNA–lncRNA–miRNA cliques. Each clique included one mRNA, one lncRNA, and one miRNA. What is more, there were three interactions between mRNA and lncRNA, mRNA and miRNA, and miRNA and lncRNA. These 7,311 mRNA–lncRNA–miRNA triplets were given in **Supplementary Material 3**. To explore the biological significances of these triplets, we did survival analysis of each RNA and calculated the log rank test p value. If a RNA’s log rank test p value was smaller than 0.05, it was listed as 1, otherwise as 0 in **Supplementary Material 3**. What is more, we performed the analysis of variance (ANOVA) among different cancer stages and calculated the ANOVA p values. If a RNA’s ANOVA p value was smaller than 0.05, it was listed as 1, otherwise as 0 in **Supplementary Material 3**. For univariate survival analysis of RNAs, there were nine mRNAs and 15 miRNAs that showed significant associations with survival but no survival related lncRNAs. For multivariate survival analysis of triplets, there were 786 triplets with log rank p values smaller than 0.05. For stage analysis, there were 637 mRNAs, 464 lncRNA, and 316 miRNAs that showed significant associations with stage.

As shown in **Table 1**, within the 7,311 mRNA–lncRNA–miRNA triplets, there were 633 coding mRNAs, 1,184 lncRNAs, and 169 miRNAs. It can be seen that about 3.80% coding mRNAs, 24.7% lncRNAs, and 81.6% miRNAs on the integrative network function through the clique structures. The miRNAs relied more on the cliques than lncRNAs. The lncRNAs may have other regulatory mechanisms, while most miRNAs were involved in sponge mechanism (Furió-Tarí et al., 2016). For mRNAs, there are many other mechanisms, such transcription factors, alternative splicing, and A-to-I RNA-editing. Furthermore, they may be regulated by lncRNAs or miRNAs alone without the complete clique structure.

**Figure 2:**
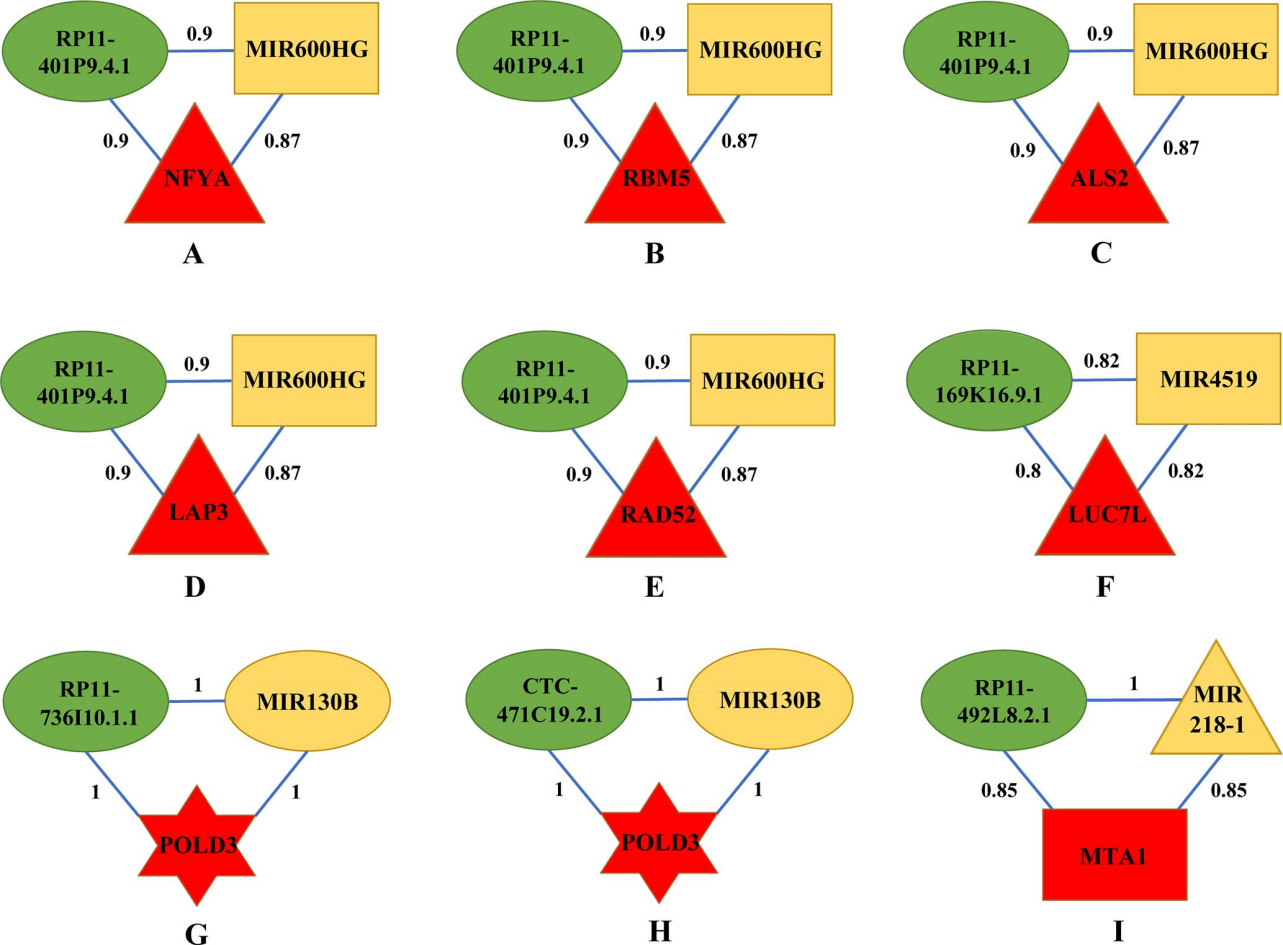
Key mRNA–lncRNA–miRNA triplets on the integrative network of ovarian cancer. The red, green, and yellow nodes represented mRNA, lncRNA, and miRNA, respectively. Each mRNA–lncRNA–miRNA triplet formed a clique on the integrative network. The rectangle, triangle, and hexagon represented survival significant, cancer-stage significant, and both significant, respectively. In a clique of mRNA–lncRNA–miRNA, there were regulations between lncRNA and mRNA, miRNA and mRNA, and lncRNA and miRNA. The numbers on the edges were adjusted R^2^. **(A)** The mRNA–lncRNA–miRNA triplets of NFYA, RP11-401P9.4.1 and MIR600HG. **(B)** The mRNA–lncRNA–miRNA triplets of RBM5, RP11-401P9.4.1 and MIR600HG. **(C)** The mRNA–lncRNA–miRNA triplets of ALS2, RP11-401P9.4.1 and MIR600HG. **(D)** The mRNA–lncRNA–miRNA triplets of LAP3, RP11-401P9.4.1 and MIR600HG. **(E)** The mRNA–lncRNA–miRNA triplets of RAD52, RP11-401P9.4.1 and MIR600HG. **(F)** The mRNA–lncRNA–miRNA triplets of LUC7L, RP11-169K16.9.1 and MIR4519. **(G)** The mRNA–lncRNA–miRNA triplets of POLD3, RP11-736I10.1.1 and MIR130B. **(H)** The mRNA–lncRNA–miRNA triplets of POLD3, CTC-471C19.2.1 and MIR130B. **(I)** The mRNA–lncRNA–miRNA triplets of MTA1, RP11-492L8.2.1 and MIR218-1.

**Figure 3:**
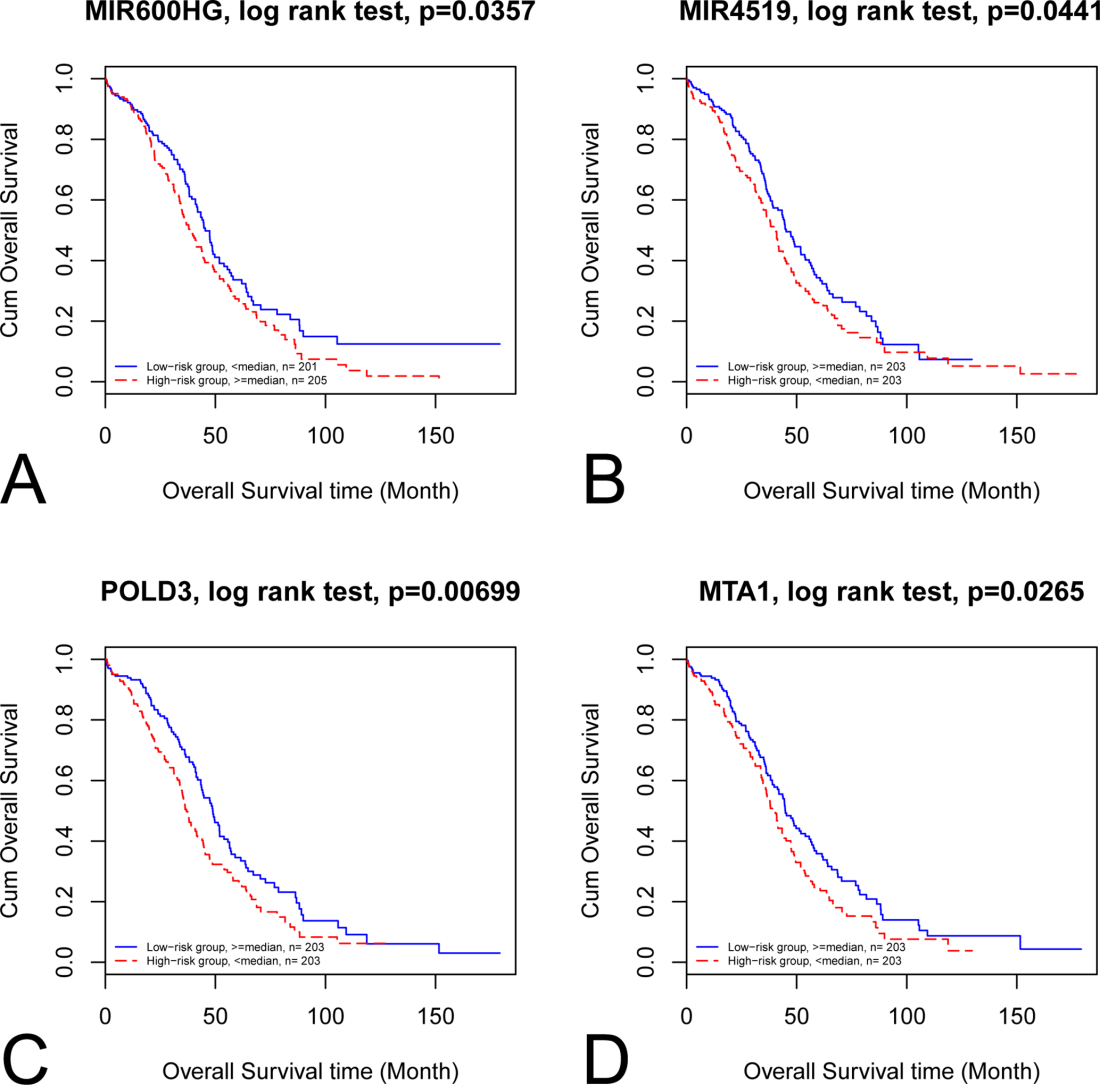
Kaplan–Meier plots of the four RNAs associated with survival. The Kaplan–Meier plots of MIR600HG, MIR4519, POLD3, and MTA1 were shown in **A**, **B**, **C**, and **D**, respectively. **(A)** The high expression of MIR600HG was associated with high risk. **(B)** The low expression of MIR4519 was associated with high risk. **(C)** The low expression of POLD3 was associated with high risk. **(D)** The low expression of MTA1 was associated with high risk.

By categorizing these mRNA–lncRNA–miRNA triplets based on survival and stage association, we plotted nine triplets that showed significant association with survival and cancer stage in **Figure 2**. The Kaplan–Meier plots of the four RNAs associated with survival, MIR600HG, MIR4519, POLD3, and MTA1, were given in **Figure 3**. The high expression of MIR600HG and the low expression of MIR4519, POLD3, and MTA1 were associated with high risk.

### Functional Analysis of Key mRNA–lncRNA–miRNA Triplets on the Ovarian Cancer Integrative Network

First, we focus on the mRNA–lncRNA–miRNA interaction and biological functions in **Figures 2A–E**, as they share the same lncRNA and miRNA. RP11-401P9.4.1, also known as lnc-NKD1-1:1, has been reported to be up regulated in multiple cancers including hepatocellular carcinoma (HCC) and clear cell renal cell carcinoma (ccRCC) (Ellinger et al., 2015; Zhang et al., 2015). Evolutionary clues in lncRNAs, especially structure, function, and expression not canonical sequences, have been validated among lncRNA homologs in vertebrate (Li et al., 2013; Xu et al., 2016; Nitsche and Stadler, 2017; Tu et al., 2018; Fico et al., 2019), underscoring the importance of conservative function in lncRNAs. *In silico* functional analysis has shown that lnc-NKD1-1:1 regulated the genes involved in mitosis and cell cycle, implying its important roles in the progression of cancer. Hence, it is reasonable to infer that lnc-NKD1-1:1 performs similar functions in ovarian cancer. MIR600HG has been proved as a prognostic biomarker in predicting survival of pancreatic ductal adenocarcinoma (PDAC) patients (Song et al., 2018), which is also consistent with our results (**Supplementary Material 3**). In addition, its function could be inferred by its significantly co-expressed gene scaffold adapter GRB2-associated binding protein 2 (GAB2), whose overexpression has been suggested to promote tumor angiogenesis and growth in ovarian cancer cells (Duckworth et al., 2016). Therefore, lnc-NKD1-1:1 may have its specific way to interact with MIR600HG for their similar contributions to promote the malignant process of cancer.

Next, we consider the five mRNAs in the triplets. NFYA, the regulatory subunit of the CCAAT binding transcription factor NFY, has been observed with elevated levels in EOC cells compared with ovarian surface epithelial cells (Garipov et al., 2013), knockdown of which suppressed growth and induced apoptosis of human EOC cells, emphasizing its requirement for ovarian cancer cell proliferation. As a component of pre-spliceosomal complexes, the RNA binding motif protein 5 (RBM5) has been demonstrated to regulate the alternative splicing process of several mRNAs (Nguyen et al., 2011). The upregulation of RBM5 has been found in ovarian and breast cancers (Farina et al., 2011), while decreased levels of RBM5 upon RAS activation were detected in various solid cancer types and exhibited its association with metastasis (Kim et al., 2010; Farina et al., 2011), suggesting its crucial roles in tumorigenesis. ALS2 encodes a ubiquitous protein Alsin, which is mainly expressed in the central nervous system (Gautam et al., 2016). It is important for maintaining cellular integrity, as mutations in ALS2 can lead to many motor neuron diseases (Yang et al., 2001). Although there is little literature about the connections between ALS2 and ovarian cancer, our results may provide new insights into the function of this gene, and further mechanisms need to be investigated. As a cell surface aminopeptidase, leucine aminopeptidase 3 (LAP3) is responsible for catalyzing the hydrolysis of leucine residues. Accumulating evidence has revealed that overexpression of LAP3 correlated with prognosis of several cancers, such as esophageal squamous cell carcinoma (ESCC), glioma, and HCC (He et al., 2015). Meanwhile, a recent study on ovarian cancer cells has indicated that suppression of LAP3 inhibited cell migration and invasion through its target genes fascin and MMP-2/9 (Wang et al., 2015b), underlying its important roles in regulating cancer cell metastasis. Previous studies have presented the involvement of radiation repair protein 52 (RAD52) in the cell cycle control as well as DNA repair, and depletion of RAD52 could cause synthetic lethality in BRCA1 mutant breast cancer cells (Hromas et al., 2017). Additionally, several groups have conferred RAD52 as novel risk loci in ovarian cancer patients (Stafford et al., 2017; Zhao et al., 2017), underlining its significant effects on controlling cancer cell activities. In conclusion, all elements in these five triplets contribute cohesively to the development of ovarian cancer through regulating cell cycle and growth.

Then, we explore the triplet mRNA LUC7L–lncRNA RP11-169K16.9.1–miRNA MIR4519 shown in **Figure 2F**. Apart from functioning in myogenesis, putative RNA-binding protein Luc7-like 1 (LUC7L) has recently been regarded as a predictor of breast cancer survival through transcriptional network construction and showed its correlation with metastasis (Crawford et al., 2008). MIR4519 contributes to tumorigenicity by interacting with its direct target N-ethylmaleimide-sensitive factor (NSF) and associated receptor SNARE. The latter have been demonstrated to be involved in cell migration and invasion by regulating vesicle trafficking and membrane fusion (Meng and Wang, 2015; Sun et al., 2016; Naydenov et al., 2018; Yoon and Munson, 2018). Furthermore, SNARE complex have been reported to affect cell apoptosis and proliferation, highlighting their great impacts on facilitating ovarian cancer cell viability (Sun et al., 2016). RP11-169K16.9.1, also called lnc-UQCRHL-1:1, is an antisense lncRNA derived from UQCRHL gene, whose genetic variants have been identified in breast cancer samples with specific DNA amplification region (Parris et al., 2018). Also, prior research has considered UQCRHL as a prognostic factor in HCC for its pivotal roles in mitochondrial respiration (Park et al., 2017). Thus, it can be inferred that lnc-UQCRHL-1:1 may exert key influences on cellular respiration, aberration of which is frequently found in tumor progression (Vyas et al., 2016). This triplet concentrates on the migration process that triggers ovarian cancer metastasis.

Subsequently, we analyze two triplets in **Figures 2G, H**, as they share the same mRNA and miRNA. Acting as a component of the DNA polymerase delta complex, POLD3 has been served as a prognostic biomarker in ovarian cancer (Willis et al., 2016), which is in accordance with our results (**Supplementary Material 3**) and indicates its possible roles in clinical application. Increasing evidence has shown that MIR130B participated in multidrug resistance in ovarian cancer by targeting NRP1 and CSF1, which influenced cell motility and adhesion (Yang et al., 2012; Chen et al., 2016). Furthermore, abnormal methylation levels of MIR130B were found in drug-resistant cell lines as well as ovarian cancer tissues (Yang et al., 2012). Although RP11-736I10.1.1 is a rare lncRNA with little literature report, its function could be speculated by its neighboring gene PLAC1, which is an X-linked trophoblast gene and has recently been identified as a serum biomarker for breast cancer (Baldwin et al., 2008; Yuan et al., 2018), suggesting its potential relationship with tumor initiation and progression. CTC-471C19.2.1, also named lnc-UBE2QL1-1:1, is originated from UBE2QL1, which has proved to interconnect with other genes enriched in cell cycle and metabolic pathways in HCC and renal cancer (Wake et al., 2013; Ye et al., 2016). Considering the aforementioned interspecies conservation in lncRNA functionality (Nitsche and Stadler, 2017; Fico et al., 2019), our results expand new links between this lncRNA and ovarian cancer. In summary, above coding and noncoding RNAs may serve as candidate genes that need more attention considering drug resistance in ovarian cancer.

The last triplet is mRNA MTA1–lncRNA RP11-492L8.2.1–miRNA MIR218-1 (**Figure 2I**). MTA1 is a metastasis-associated gene and has been observed to stimulate proliferation of EOC cells by virtue of enhancing DNA repair (Yang et al., 2014). Previous studies have revealed that MIR218-1 affected tumor angiogenesis and further has specific influences on cell migration and invasion in several cancers including cervical squamous cell carcinoma and prostate cancer (Yamamoto et al., 2013; Guan et al., 2016), stressing its great effects on determining cancer cell fate. RP11-492L8.2.1, also annotated as lnc-DMXL1-7, may interact with DMXL1, whose upregulation was found in leukemic cells (Koldehoff et al., 2008). Given these interactions, these three RNAs cluster together for their similar efforts to tumor initiation and progression mediated by regulating angiogenesis, proliferation, and migration of malignant cells.

### Identification of Four Cliques Based on Network Structure Analysis

We have analyzed the mRNA–lncRNA–miRNA triplets, which were actually three cliques. If we increase the size of cliques to four, what will happen? To investigate the effects of clique size, we extracted the four cliques from the integrative network. There were 11,117 four cliques with at least one mRNA, lncRNA, and miRNA. The number of four cliques was greater than the number of triplets, which was 7,311. It suggested that some four cliques shared the same triplets. The 11,117 four cliques containing mRNA–lncRNA–miRNA triplet were given in **Supplementary Material 4**. To explore the biological significances of these four cliques, we also did survival analysis and ANOVA for each RNA. If a RNA’s log rank test p value was smaller than 0.05, it was listed as 1, otherwise as 0 in **Supplementary Material 4**. If an RNA’s ANOVA p value among different cancer stages was smaller than 0.05, it was listed as 1, otherwise as 0 in **Supplementary Material 4**.

**Figure 4:**
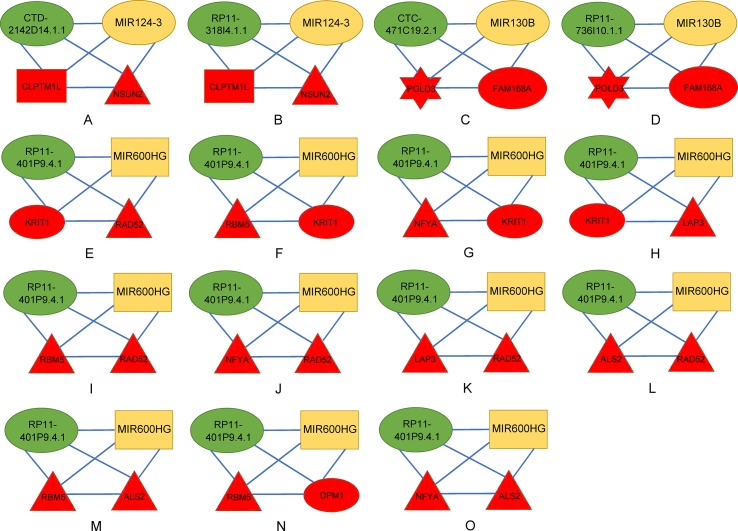
Four-cliques that were both associated with survival and cancer stage. The red, green, and yellow nodes represented mRNA, lncRNA, and miRNA, respectively. The rectangle, triangle, and hexagon represented survival significant, cancer-stage significant, and both significant, respectively. **(A)** Four-cliques of CTD-2142D14.1.1, MIR124-3, CLPTM1L and NSUN2. **(B)** Four-cliques of RP11-318I4.1.1, MIR124-3, CLPTM1L and NSUN2. **(C)** Four-cliques of CTC-471C19.2.1, MIR130B, POLD3 and FAM168A. **(D)** Four-cliques of RP11-736I10.1.1, MIR130B, POLD3 and FAM168A. **(E)** Four-cliques of RP11-401P9.4.1, MIR600HG, KRIT1 and RAD52. **(F)** Four-cliques of RP11-401P9.4.1, MIR600HG, RBM5 and KRIT1. **(G)** Four-cliques of RP11-401P9.4.1, MIR600HG, NFYA and KRIT1. **(H)** Four-cliques of RP11-401P9.4.1, MIR600HG, KRIT1 and LAP3. **(I)** Four-cliques of RP11-401P9.4.1, MIR600HG, RBM5 and RAD52. **(J)** Four-cliques of RP11-401P9.4.1, MIR600HG, NFYA and RAD52. **(K)** Four-cliques of RP11-401P9.4.1, MIR600HG, LAP3 and RAD52. **(L)** Four-cliques of RP11-401P9.4.1, MIR600HG, ALS2 and RAD52. **(M)** Four-cliques of RP11-401P9.4.1, MIR600HG, RBM5 and ALS2. **(N)** Four-cliques of RP11-401P9.4.1, MIR600HG, RBM5 and DPM1. **(O)** Four-cliques of RP11-401P9.4.1, MIR600HG, NFYA and ALS2.

**Figure 5:**
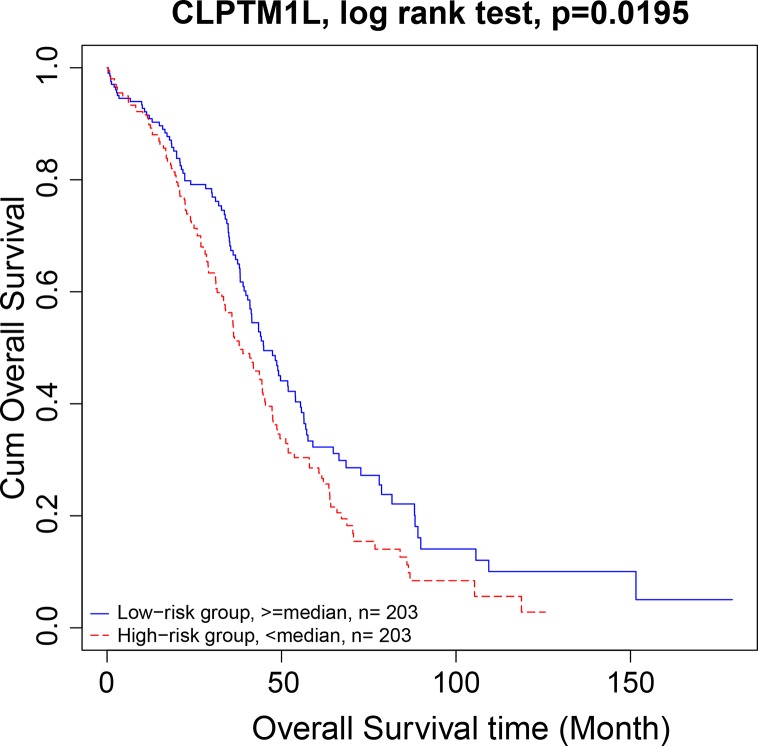
Kaplan–Meier plot of CLPTM1L. The high expression of CLPTM1L was associated with high risk.

There were 15 four cliques that were both associated with survival and cancer stages (**Figure 4**). The survival-associated RNAs in these four cliques were MIR600HG, POLD3, and CLPTM1L. MIR600HG and POLD3 have already been discussed. The Kaplan–Meier plots of CLPTM1L were given in **Figure 5**. The high expression of CLPTM1L was associated with high risk.

We then move on to the functional analysis of a few new genes in four cliquesas other genes have been discussed above (**Figures 4I, J, K, L, M and O**). **Figures 4A, B** shares the same mRNAs and miRNA. Cleft lip and palate transmembrane protein 1-like (CLPTM1L) was frequently found to be up regulated in cisplatin-resistant human ovarian cancer cells (Yamamoto et al., 2001; James et al., 2012) and also associated with cisplatin-induced apoptosis. Recent study has uncovered the role of CLPTM1L in modulating cell survival signaling and identified it as a lung cancer risk gene (James et al., 2012; James et al., 2014), which was also validated by our results (**Figure 5**). The tRNA methytransferase NSUN2 has been shown to stimulate cell growth in a cell cycle-dependent manner due to its dynamic expression levels throughout the cell cycle (Xing et al., 2015), suggesting its great roles in promoting tumorigenesis. Accumulating studies have revealed the function of MIR124-3 in inhibiting invasion and migration of malignant cells both in ovarian cancer and hepatocellular cancer (Zhang et al., 2013; Cai et al., 2017; Yuan et al., 2017). Although the roles of two lncRNAs CTD-2142D14.1.1 and RP11-318I4.1.1 have not been elucidated yet, it can be deduced by their significant co-expression with CLPTM1L and NSUN2 from TCGA ovarian cancer data, indicating their cooperation in contributing to the progression of ovarian cancer. As for **Figures 4C and D**, FAM168A, also known as tongue cancer resistance-related protein 1(TCRP1), has previously been found to mediate resistance to chemotherapy in several cancers including ovarian and lung cancers (Gu et al., 2017; Liu et al., 2017b), exerting similar function to the triplet in **Figure 2H**, which explained their clusters and emphasized their importance on drug resistance in ovarian cancer. Besides, FAM168A has been identified as a prognostic marker in renal cancer, endometrial cancer, and urothelial cancer (Gu et al., 2017). The new gene in **Figures 4E, F, G, H** is Krev interaction trapped protein 1 (KRIT1), which is responsible for cerebrovascular disease (Goitre et al., 2010). Consistent with other partners in this four cliques, KRIT1 has been shown to be involved in cell growth, DNA damage, angiogenesis, and cell death (Goitre et al., 2010; Orso et al., 2013), indicating that it may function as a potential mechanism of susceptibility to ovarian tumorigenesis. **Figure 4N** contains a new gene DPM1 that encodes dolichol-phosphate mannosyltransferase. Previous work has examined increased expression levels of DPM1 in primary EOC of various histotypes (Ramakrishna et al., 2010). Moreover, DPM1 has been considered as a prognostic marker in liver cancer. Altogether, these new genes in four cliques are capable of undertaking ovarian cancer risk candidate genes on account of their contributions to the development of ovarian cancer.

### Identification of the Common Clique Between Ovarian Cancer and Uterine Corpus Endometrial Carcinoma

In our previous paper (Liu et al., 2017a), we have identified the cliques in uterine corpus endometrial carcinoma (UCEC). Both uterine corpus endometrial carcinoma and ovarian cancer were common female cancers. We compared the cliques in ovarian cancer with the cliques in UCEC and found one overlapped clique: mRNA KRTAP24-1–lncRNA LL22NC03-121E8.3.1–miRNA MIR2116. KRTAP24-1, also called KAP24.1, is a cuticular hair keratin-associated protein (KAP) and belongs to human KAP family because of its structure and location (Rogers et al., 2007). More than as the intermediate filament forming proteins of epithelial cells, keratins have recently been regarded as regulators of protein synthesis, cell motility, and membrane traffic and signaling (Karantza, 2011). Furthermore, keratins have been extensively involved in cancer cell invasion and metastasis as well as acted as diagnostic markers in epithelial cancers (Moll et al., 2008), suggesting its vital roles in the development of cancer. MIR2116 is a newly found miRNA expressed in EOC (Wyman et al., 2009) and associated with bacterial infection (Zhao et al., 2018; Modak et al., 2019). Our results expand its function in the pathogenesis of gynecological cancers. Although LL22NC03-121E8.3.1 is a novel lncRNA with little reports, we can speculate its function by its interacted protein fragile X mental retardation 1 (FMR1), which is expressed at high levels and considered as a prognostic marker in gynecological cancers including ovarian and endometrial cancers (Hagerman et al., 2017), indicating its potential roles in facilitating tumorigenesis. More recently, several lines of evidence have supported that the estrogen receptor (ER) was highly expressed in ovarian cancers and regulated cell growth, which attracted endocrine therapy for ovarian cancer treatment (Lee et al., 2014; Langdon et al., 2017). Meanwhile, the main histopathologic type of endometrial cancer (type I) is strongly related to unopposed estrogen (Lee et al., 2014), and hormones represent a promising target especially in advanced or recurrent endometrial cancer. Taken these into account, hormones become common features in the progression of ovarian and endometrial cancers. As this triplet is both found in OC and UCEC, it is reasonable to infer that these three RNAs have a strong relationship with hormone production through regulating their expression and stability, which needs more investigations. In addition, our results provide new hormone-related RNAs about gynecological oncogenesis.

## Conclusion

The regulation roles of lncRNAs and miRNAs are largely unknown. Recent studies have shown that lncRNAs and miRNA can cross talk and create a competition for binding between miRNA, lncRNA, and regulatory target, which is called sponge effect. To investigate the functional relationship between miRNA, lncRNA, and mRNA on a genome-wide scale is essential for understanding the regulatory roles of lncRNA and miRNA. Therefore, we analyzed the RNA-seq profiles of 407 ovarian cancer patients and constructed an integrative network of 20,424 coding mRNAs, 10,412 lncRNAs, and 742 miRNAs using VIF regression. Then, all the mRNA–lncRNA–miRNA cliques were detected using R package *igraph*. Specifically, we analyzed the triplets that showed significant correlations with survival and stage of ovarian cancer. Our results not only provide novel insights of the regulatory mechanisms among mRNAs, lncRNAs, and miRNAs, but also shield light on the tumorigenesis mechanisms of ovarian cancer.

## Author Contributions

YZ and JJ conceived of the study and participated in its design and coordination. XZ and WH conducted the database search and analysis. TH and BX conducted the network construction. YZ conducted the biological analysis and wrote the first draft. The draft was improved through discussion and editing by all the authors, who read and approved the final manuscript.

## Funding

This study was supported by the National Key Technology Support Program of China (2015BAI12B00), National Key R&D Program of China (2018YFC0910403), National Natural Science Foundation of China (31701111, 31701151), Shanghai Municipal Science and Technology Major Project (2017SHZDZX01), China Postdoctoral Science Foundation (2018M642305), Natural Science Foundation of Jiangsu Province (BK20170295), Youth Innovation Promotion Association of Chinese Academy of Sciences (2016245), and Basic Research Project of Changzhou (CJ20179024).

## Conflict of Interest Statement

The authors declare that the research was conducted in the absence of any commercial or financial relationships that could be construed as a potential conflict of interest.

## Supplementary Materials

Supplementary Material 1 The TCGA sample IDs and their clinical information.

Supplementary Material 2 Constructed integrative network.

Supplementary Material 3 mRNA–lncRNA–miRNA triplets.

Supplementary Material 4 Four-cliques containing mRNA–lncRNA–miRNA triplets.
